# Upcycling Walnut Green Husk: Polyphenol-Rich Extracts from Traditional vs. Organic Crops for Spray-Dried Vegan Additive Development

**DOI:** 10.3390/polym17172371

**Published:** 2025-08-31

**Authors:** Silvia Matiacevich, Ignacio Durán, Marlen Gutiérrez-Cutiño, Javier Echeverría, César Echeverría, Daniela Soto-Madrid

**Affiliations:** 1 Food Properties Research Group (INPROAL), Department of Food Science and Technology, Technological Faculty, Universidad de Santiago de Chile, Santiago 9170201, Chile; ignacio.duran@usach.cl; 2 Instituto de Alta Investigación, Universidad de Tarapacá, Arica 1020000, Chile; marlen.gutierrez@usach.cl; 3 Departamento de Ciencias del Ambiente, Facultad de Química y Biología, Universidad de Santiago de Chile, Santiago 9170022, Chile; javier.echeverriam@usach.cl; 4 Instituto de Ciencias Naturales, Facultad de Medicina Veterinaria y Agronomía, Universidad de Las Américas, Santiago 7500590, Chile; cesar.echeverria@uda.cl; 5 Centro de Investigación en Ciencias Biológicas y Químicas, Universidad de Las Américas, Santiago 7500975, Chile; 6 Molecular Biology and Genetics Laboratory, Faculty of Medicine, ATACAMA-OMICS, Copiapó 1530000, Chile; 7 Department of AgroIndustry and Enology, Facultad de Ciencias Agronómicas, Universidad de Chile, Santa Rosa 11315, Santiago 8820808, Chile

**Keywords:** upcycling, walnut green husk, polyphenols, additive

## Abstract

This study explores the valorization of walnut green husk, an agro-industrial by-product, through ultrasound-assisted extraction to obtain polyphenol-rich extracts with antioxidant properties. The extracts demonstrated non-cytotoxicity, regardless of the presence of pesticides, antibiotics, or the type of crop. Notably, organic walnut husk yielded higher total polyphenols and antioxidant activity, identifying 37 polyphenolic compounds compared to 22 in traditional crops. Chickpea protein was utilized as a wall material to encapsulate the extract, resulting in a sustainable, vegan antioxidant powder. Optimal results were achieved using 5% (*w*/*v*) chickpea protein and spray drying at 136 °C, yielding a light-colored powder with high antioxidant content and stability under low humidity (≤35%). The product shows promise as a natural, plant-based alternative to synthetic antioxidants in food systems. Further studies are needed to evaluate its functional and technological performance during food integration and storage.

## 1. Introduction

The growing environmental impact of agro-industrial waste has increased global interest in sustainable strategies aligned with the principles of the circular economy, which emphasize the recovery and valorization of by-products [1,2]. In parallel, increasing consumer demand for clean-label, plant-based, and environmentally responsible ingredients is driving innovation in the food industry toward the use of natural additives with proven functional benefits, supporting both health-conscious dietary trends and ecological sustainability. A preliminary life cycle and economic feasibility assessment indicates that this valorization strategy provides environmental and economic advantages over conventional alternatives for additive production and waste management [2].

Chile, as one of the world’s leading walnut exporters, generates approximately 180,000 tonnes of walnuts annually. Of this production, an estimated 20% comprises green walnut husks [3]—a by-product typically discarded, despite its potential as a rich source of bioactive compounds. Previous studies have identified this material as particularly abundant in polyphenols, with promising antioxidant and antimicrobial properties [4,5,6]. Various extraction methods and process conditions have recently been employed to extract polyphenols from walnut green husk, including the traditional method of maceration and ultrasound-assisted extraction (UAE) [5,7,8]. UAE uses solvents such as water, ethanol, or an ethanol-water mixture, which is important due to its simplicity, efficiency, and sustainability. It enables solvents to penetrate cells more effectively, reduces extraction times, and increases the yield of phenolic compounds extracted from the vegetal matrix (e.g., walnut green husks), as demonstrated by Soto-Madrid [5].

The recovery of polyphenol-rich extracts from walnut green husks represents a valuable opportunity to address two pressing challenges: the need for sustainable waste valorization and the development of natural food ingredients. These extracts, with their established health-promoting potential [6,9], could not only enhance the nutritional value of food products but also contribute to extending their shelf life, offering a natural and sustainable alternative for the formulation of next-generation food additives.

Polyphenol-rich extracts offer significant potential as natural food additives; however, their application is limited due to their instability when exposed to light, oxygen, and humidity [9]. Encapsulation, particularly through spray drying, is a widely recognized and cost-effective strategy for improving compound stability and enabling the development of powdered ingredients with extended shelf life and preserved functionality [9,10].

Therefore, the selection of appropriate wall materials is crucial for controlling encapsulation efficiency, physicochemical properties, and the storage stability of microencapsulated powders [11]. Proteins (sodium caseinate, whey proteins, soy proteins, chickpea proteins, and gelatin) and carbohydrates (modified starch, gum arabic, corn syrup, and maltodextrin) are the most common wall materials used in microencapsulation, due to their biocompatibility, biodegradability, and good amphiphilic and functional properties, such as water solubility and emulsifying capacity. For that, proteins are ideal for encapsulating both hydrophobic and hydrophilic substances [12,13], and have proven to be effective and emerging encapsulating agents for the ability to form protective matrices around sensitive compounds [14,15]. Among them, chickpea protein stands out as a sustainable, plant-based option with good solubility, low allergenicity, and desirable functional properties for vegan product development [16,17,18]. Its ability to interact with polyphenols and form stable complexes [19] enhances protection under storage conditions [20,21].

It is especially relevant for walnut green husks, whose color and bioactivity are attributed to compounds such as naphthoquinones, flavonoids, and tannins [5], which are highly sensitive to moisture and oxygen (Galanakis, 2018 [9]). High humidity accelerates oxidation, particularly of juglone, resulting in the formation of dark pigments and undesirable color changes [15,22]. Chickpea protein can mitigate this effect by acting as a barrier, stabilizing color, and protecting compounds through the formation of three-dimensional networks [21].

Despite the recognized potential of walnut husk polyphenols, it is essential to evaluate the composition and safety of extracts obtained from different agricultural practices. Residues from traditional crops may contain pesticide traces, posing cytotoxic risks [23], highlighting the need for proper safety assessments in value-added applications.

The rising popularity of veganism and plant-based diets is reshaping both sustainable consumption habits and the food industry [24,25]. As a result of these market changes, there is a growing need for new ingredients and additives to meet the demands of these plant-based products.

Therefore, this study aimed to evaluate the potential of polyphenol-rich extracts from walnut green husks, sourced from both traditional and organic crops, for use in spray-dried vegan additives. The research involves the chemical and physicochemical characterization of the by-products and the polyphenolic identification of extracts, the optimization of encapsulation with chickpea protein, and an assessment of storage stability, thereby contributing to the sustainable and safe use of agro-industrial by-products in food systems.

## 2. Materials and Methods

### 2.1. Samples

Green husks from *Juglans regia* cv. Chandler (Juglandaceae) were manually harvested at full maturity in April 2023 (fall), before the rainy season, from six-year-old trees with completely open husks in a central 10 × 10 tree plot, thereby avoiding edge effects. Two types of samples were obtained. The first was an organic crop sample (Geonuts, Metropolitan Region, Chile), certified as organic management, located in San Bernardo (33°38′21.892″ S, 70°41′55.563″ W, 581 AMSL). This soil belongs to the Entisols order [26] and is located southwest of Santiago in an area with a temperate-warm continental climate. The second sample originated from a traditional crop in a conventional plantation, harvested in Cuncumén, Valparaíso Region, Chile (33°43′58″ S, 71°25′37″ W, 182 AMSL) [15]. This area is situated in the coastal mountain range of the Valparaíso region, characterized by a humid Mediterranean coastal climate. This soil belongs to the Mollisols order. Both soil types support much of the irrigated agriculture in central Chile [26]. A location map is included in the Supplementary Materials (Supplementary Figure S1).

Postharvest, husks were dried in a forced-air oven at 40 °C for 48 h (Zenith lab, DHG-9053A, Jiangsu, China), ground using a Thermomix (Vorwerk, Wuppertal, Germany), and stored in glass containers protected from light at room temperature until extraction.

Chickpea protein (CPP) used as an encapsulating agent was extracted from defatted chickpea flour (Extrumol, Santiago, Chile) via alkaline solubilization and isoelectric precipitation, following the method described by Soto-Madrid [27]. The resulting protein was freeze-dried for 48 h (Ilshin FD5508, Dongducheon, Republic of Korea) at a tray temperature of 40 °C and chamber temperature of −60 °C. CPP presented 4.20 ± 0.01 (g/100 g dry base) of moisture and a protein concentration percentage of 85 ± 7 (g/100 g dry base).

### 2.2. Raw Material and Extract Characterization

A proximate analysis was performed on the walnut green husk, considering both organic and traditional samples, as well as on the aqueous extract and the protein concentrate. This analysis determined the moisture, protein, lipid, carbohydrate, ash, crude fiber, and nitrogen-free extract contents, following the methodologies of the Association of Official Analytical Chemists [28].

Additionally, the water activity of the walnut green husk was measured using an Aqualab (Aqualab, Serie 3 TE, Pullman, WA, USA), both before and after oven drying.

### 2.3. Ultrasound-Assisted Extraction

Polyphenol extraction was performed using probe sonication (Sonics Materials, VCX 500, Newtown, PA, USA), employing a mixture of ethanol and water as the solvent in a 75:25 (*v*/*v*) ratio and a solid-to-solvent ratio of 1:25 (*w*/*v*). Crushed walnut green husk was used as the solid material, according to the methodology of Soto-Madrid [5]. The extract was then filtered using a vacuum pump (Rocker, model 300 C, New Taipei, Taiwan) and Whatman 1 filter paper. Finally, the ethanol was evaporated in a rotary evaporator (Büchi R-100, Flawil, Switzerland) at 40 °C. The obtained aqueous extract was stored under refrigeration and protected from light until further analysis was performed.

### 2.4. Determination of Total Polyphenol Content (TPC)

The total polyphenol content (TPC) was determined using the Folin–Ciocalteu method [29] for both the extract and the obtained powder. Before analysis, the powder was reconstituted to a 1% (*w*/*v*) concentration and shaken with purified water on a magnetic stirrer at 490 rpm for 20 min. Gallic acid of analytical grade (Merck, Darmstadt, Germany) was used to prepare the calibration curve (0–0.8 mg/mL). The results were expressed as milligrams of Gallic Acid Equivalents (GAE) per gram of dry sample.

### 2.5. Determination of Antioxidant Capacity by DPPH

The 2,2-diphenyl-1-picrylhydrazyl (DPPH) free radical inhibition method measures the concentration of the antioxidant compound necessary to reduce the concentration of DPPH, following the methodology of Brand-Williams [30]. The antioxidant capacity was evaluated in both the extract and the powder, which had been previously reconstituted to 5% (*w*/*v*). Trolox (Sigma-Aldrich, Buchs, Switzerland) was used as a reference standard to create the calibration curve (0–400 mg/L). The results were expressed in milligrams of Trolox per gram of dry sample.

### 2.6. Compounds Identification by UPLC-QTOF-ESI-MS

The samples, including both the extracts, were analyzed using ultra-performance liquid chromatography quadrupole time-of-flight electrospray ionization mass spectrometry (UPLC-QTOF-ESI-MS Waters Xevo G2-XS QTof/Tof, Milford, MA, USA) with a column (1.8 μm; 2.1 × 100 mm) maintained at 30 °C and an injection volume of 5 μL, utilizing a gradient over 3 to 40 min with a constant flow rate of 0.2 mL/min.

Mobile phase A consisted of water with 0.1% (*v*/*v*) formic acid, while mobile phase B was 100% acetonitrile (Merck, Darmstadt, Germany). The gradient for solvent A was 100% (0–3 min), 10% (30–33 min), and 100% (35–40 min), and for solvent B was 0% (0–3 min), 90% (30–33 min), and 0% (35–40 min). The ionization source was electrospray ionization (ESI), and the mass spectrum was detected at 40 min in negative ion mode, with a mass range of 50–1200 *m*/*z*. The desolvation gas temperature was 300 °C, with a gas flow of 500 L/h, a collision energy of 10 V (low energy), and 10–75 V (high energy).

The mass spectrometer also operated in both positive and negative ion modes. Identification was performed using Progenesis QI MetaScope software v3.1 (Waters, Milford, MA, USA), with search parameters derived from the spectral library of the Human Metabolome Database (HMDB). The precursor ion and fragments were analyzed with an error tolerance of 10 mg/kg. All compounds found were reported to have adduct formation in the negative M-H mode. Subsequently, the results were filtered using a fragmentation score greater than 80% and classified through the PubChem (https://pubchem.ncbi.nlm.nih.gov/) and NPClassifier (https://npclassifier.ucsd.edu/) websites (accessed on 23 June 2025) [31].

### 2.7. In Vitro Cell Viability and Oxidative Stress of Extracts

A cell viability assay by resazurin was performed to determine the cytotoxicity of the natural extracts (organic and traditional), following the methodology described by Mitsi [32]. The cytotoxic properties were assessed using 5 × 10^3^ Vero cells (a non-tumor cell line (American Type Culture Collection, ATCC, CCL-81, Santiago, Chile)) seeded per well in 96-well microplates. The hydroethanolic extract of walnut green husks was resuspended in water at a concentration of 10 mg/mL. Subsequently, 20 μL of a resazurin solution (0.5 mg/mL in phosphate-buffered saline, PBS) was added to each well, and the plates were incubated at 37 °C for 4 h in a humidified atmosphere with 5% CO_2_. After incubation, the wells were analyzed for fluorescence using a Tecan reader (Infinite 200 Pro, Männedorf, Switzerland), with an excitation wavelength of 560 nm and an emission wavelength of 590 nm. Ellipticine was used as the positive control, while each cell suspension served as the negative control. The results were expressed in CC_50_ values (concentration that inhibited 50% of the cell proliferation). Three independent assays were performed in triplicate.

Intracellular reactive oxygen species (ROS) detection was performed using the 2′,7′-dichlorofluorescein diacetate (DCFH-DA) assay. The methodology was followed as reported by Mitsi [32]. The cell line used was the human hepatocellular carcinoma cell line Hep-G2 (ATCC HB-8065, Atacama, Chile). Exponentially growing cells were seeded in black-walled 96-well plates at a density of 10 × 10^3^ cells per well and incubated overnight with 100 μL of culture medium per well. The culture medium was replaced with 100 μL of fresh medium without fetal bovine serum (FBS) containing 10 μM DCFH-DA. This probe permeates live cells, where intracellular esterases hydrolyze DCFH-DA to DCFH. Upon exposure to ROS, DCFH is oxidized to DCF, a fluorescent compound that can be detected and quantified. The intensity of the fluorescent signal is directly proportional to the intracellular ROS levels. After 30 min of incubation with DCFH-DA, the solution was removed, and cells were treated with different extract concentrations in 100 μL of FBS-free culture medium per well. Fluorescence was measured using a TECAN Infinite 200Pro plate reader (Tecan Trading AG, Männedorf, Switzerland) at excitation and emission wavelengths of 495 nm and 525 nm, respectively, over 4 h, with readings taken every 5 min. Intracellular ROS levels were expressed as relative fluorescence units (RFU). A 100 μM CuSO_4_ solution was used as a positive control. Untreated cells were used as a negative control. Statistical analysis was performed by calculating the change in RFU values 4 h after extract addition compared to the initial measurement (ΔRFU = RFU_4_h − RFU_0_h). Graphs were generated using GraphPad Prism version 10.2.2 (GraphPad Software, La Jolla, CA, USA).

### 2.8. Encapsulant Concentration by Spray Drying

A factorial design was used to determine the protein concentration (*w*/*v*). Three protein concentrations (5%, 7.5%, and 10% *w*/*v*) were evaluated using spray drying (Büchi B-290, Flawil, Switzerland) at the following conditions (previously determined), inlet temperature of 136 °C, pump speed at 10% (3 mL/min feed flow), nozzle air flow of 600 L/h, and aspiration set at 90% (35 m^3^/h). The response variables were TPC (as described in Section 2.4), antioxidant capacity measured using the DPPH method (as described in Section 2.5), encapsulation efficiency, drying yield, moisture content, and color.

#### 2.8.1. Encapsulation Efficiency

The encapsulation efficiency was calculated based on the TPC value of the powder additive and the extract, following the methodology of Soto-Madrid [15]. To determine the encapsulation efficiency, the following equation was used:

(1) $$Encapsulation\;efficiency\;\left(\%\right)\;=\;\frac{TPCb\;-\;TPCp}{TPCp}\;*\;100$$

where

*TPCb*: corresponds to the value of the total polyphenol content of the sample before drying;

*TPCp*: corresponds to the value of total polyphenol content of the powder additive.

#### 2.8.2. Drying Yield

The total soluble solids (TSS) of the extract with encapsulating agent (TSS/100 mL of extract) were measured using refractometry (Hanna, HI96800, Limena, Italy) before the drying process, referred to as initial solids. After drying, the obtained powders were weighed on an analytical balance (A&D HR-120, Milpitas, CA, USA) to determine the total dry solids. Based on these values, the drying yield (DY) was calculated.

(2) $$Drying\;Yield\;\left(\%\right)=\frac{Total\;Solids\;Dring}{Total\;Solids\;Before\;Drying\;}\times 100$$

#### 2.8.3. Water Activity and Moisture Content

The moisture content of the powdered additive was determined gravimetrically by measuring the difference in mass before and after drying the samples in an oven (Shel Lab 1410-2E, Cornelius, OR, USA) at 105 °C until constant weight was reached, following the AOAC methodology [28]. The results were expressed as a percentage (g powder/100 g water). Water activity was measured using the method described in Section 2.2.

#### 2.8.4. Color Determination

The color of the powder was determined using image analysis with a previously calibrated computer vision system, as described by Matiacevich [33]. Digital color parameters were obtained from the image in the RGB (Red, Green, Blue) color space using Adobe Photoshop v7.0 (Adobe Systems Incorporated 2007, San José, CA, USA). The CIELab* color space values were calculated from these values, where the L* parameter indicates lightness, a* indicates the red-green axis, and b* indicates the blue-yellow axis. Color difference was calculated using the CIEDE2000 color-difference formula [34]. To analyze the powder’s color, it was compressed into a tablet using a manual press (Quick Press, Perkin-Elmer, Waltham, MA, USA).

### 2.9. Stability Analysis at Different Relative Humidities

The spray-dried powder was stored at room temperature (25 °C) and exposed to different relative humidities (RH) using desiccators with supersaturated solutions of MgCl_2_ (35% RH) and NaCl (80% RH), following the methodology described by Greenspan [35].

Moisture content and water activity were determined at different time intervals. Additionally, the color of the powder was evaluated using previously described methodologies (Section 2.8.4).

### 2.10. Physicochemical Properties of the Vegan Powdered Additive

#### 2.10.1. Total Polyphenol Content and Antioxidant Capacity of Powders

The powder was reconstituted to a 1% (*w*/*v*) concentration and stirred with purified water using a magnetic stirrer at 490 rpm for 20 min. Total polyphenol content and antioxidant capacity were determined using the previously mentioned methods (Section 2.4 and Section 2.5, respectively).

#### 2.10.2. Morphology Powder

The morphology of the vegan powder additive was characterized using a ZEISS EVO MA10 scanning electron microscope (SEM) (Zeiss, Munich, Germany), operating at an accelerating voltage of 20 kV. The samples were coated with a thin layer of gold to prevent charging effects during measurements. Additionally, the SEM is equipped with an Oxford X-Act energy-dispersive spectrometer (EDS) (Oxford Inst. Abindong, UK), which is used for quantitative elemental analysis through point and area scan modes.

### 2.11. Statistical Analysis

Statistical analysis was performed using analysis of variance (ANOVA) with a *p*-value < 0.05 to evaluate significant differences between the samples. The results were reported as the mean of three replicates, along with their standard deviation. Data was processed using the Statgraphics Centurion XVI (version 16.1.03) and GraphPad Prism (version 10.5.0).

## 3. Results and Discussion

### 3.1. Characterization of Raw Material and Extracts

The walnut green husk obtained from an organic crop was characterized through proximal analysis of the dry raw material and aqueous extract, and the results were compared with those of a sample obtained from a traditional crop (Table 1).

When comparing the proximal analysis of a traditional sample of walnut green husk, the results are similar to those previously reported by Soto-Madrid [5] and Vazquez-Flores [37]. The slight differences between them could be attributed to the fields, year of harvest, type of agricultural soil, and climate of the samples. Notably, the low moisture content of the husk is beneficial as it minimizes oxidative and enzymatic reactions that could degrade the active compounds, specifically polyphenols [38].

As expected, proximate analysis of the extracts showed a high moisture content (~94%), consistent with their aqueous nature. The absence of proteins, lipids, and fiber confirms the absence of contamination during extraction, highlighting the efficiency and selectivity of the methodology employed. Characterizing the raw material is crucial for standardizing the extraction process, as its proximate composition may vary depending on several factors. Furthermore, the absence of interfering compounds, such as lipids or proteins, is particularly important to ensure the encapsulation efficiency of phenolic compounds.

The TPC, antioxidant capacity, and physical properties were characterized in two fields: a traditional crop harvested in 2021, and an organic crop harvested in 2023.

**Figure 1:**
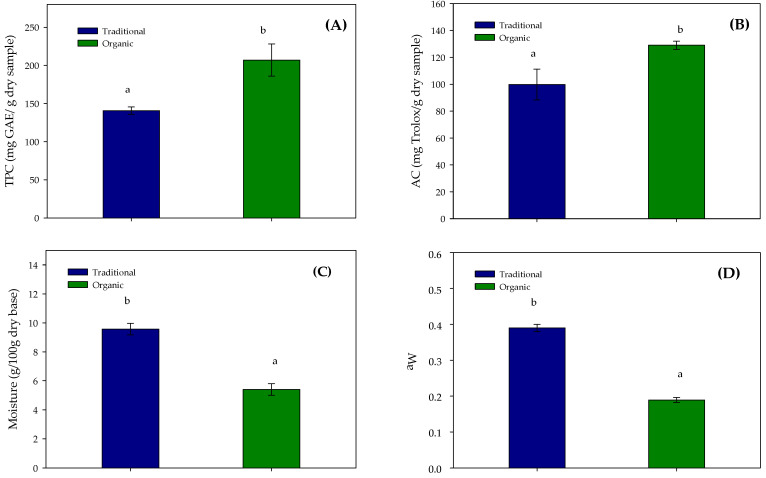
Comparison of traditional (2021) and organic harvest (2023) in physicochemical parameters: Total polyphenol content (TPC) (**A**), Antioxidant capacity (AC) measured by DPPH (**B**), Moisture (**C**), and Water activity (a_w_) (**D**). Different letters (a, b) indicate significant differences (*p* < 0.05) between the samples each year. Error bars represent the mean with the respective standard deviation of three replicas.

As shown in Figure 1A,B, the 2023 walnut husk harvest exhibited higher total polyphenol content (TPC = 207 ± 21 mg GAE/g dry sample) and antioxidant capacity (AC = 129 ± 3 mg Trolox/g dry sample) than the 2021 harvest.

These results are in agreement with Barański [39], who reported that organic crops generally contain higher polyphenol levels than traditional crops. It is important to note that although the 2021 sample was analyzed in 2023, no significant differences (*p* > 0.05) were found in TPC and antioxidant capacity compared to their initial values [5], confirming the good stability of polyphenols over a 2-year storage period.

Moisture and water activity data (Figure 1C,D) indicate the stability of the samples. The 2023 sample had significantly lower moisture (5.4% ± 0.4%) than the 2021 sample (9.0% ± 0.5%), attributed to the prolonged storage. Despite this, both samples maintained a water activity value below 0.4, confirming microbiological safety for at least two years under room-temperature and dark conditions.

### 3.2. Tentative Identification of Compounds by UPLC-QTOF-ESI-MS

The antioxidant capacity of walnut green husk is largely attributed to its diverse polyphenolic content. To tentatively identify these compounds, aqueous extracts were analyzed by UPLC-ESI-QTOF-MS. Identification was based on fragmentation scores above 80 and cross-referenced with previously reported walnut husk phytochemicals [40]. The detected compounds included hydrolysable tannins, naphthoquinones, phenolic acids, flavonoids, triterpenoids, and others, which were grouped into chemical superclasses (e.g., flavonoids, lignans, and carotenoids). Full compound data are provided in the Supplementary Table S1. The profile of traditional extracts had been previously reported by Soto-Madrid [15].

**Figure 2:**
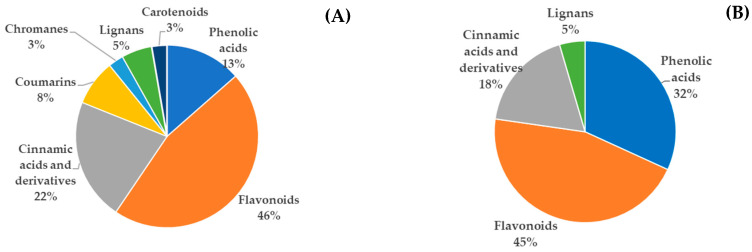
Classification of the phenolic compounds identified in the aqueous extract of the walnut green husk from (**A**) organic crop samples (37 phenolic compounds) and (**B**) traditional crop samples (22 phenolic compounds).

A total of 37 phenolic compounds were identified in extracts from organic crops, compared to 22 in traditional samples (Figure 2).

Both extract types contained hydroxybenzoic acids, including gallic acid—identified by its typical *m*/*z* fragmentation patterns [41,42]—as well as protocatechuic and caffeic acids [5,43]. Flavonoids, which represent a significant portion of polyphenols in plants [44], were abundant in both sources. Catechin and kaempferol were notably detected in both crop types [5,43], consistent with their known role in plant defense mechanisms [45]. Interestingly, compounds such as coumarins, chromanes, and carotenoids were also tentatively found in the organic crop. At the same time, a greater proportion of phenolic acids was observed in the traditional samples, potentially reflecting differences in agricultural practices and pesticide use [46]. The flavonoid profile, however, remained comparable across both conditions.

Overall, the majority of identified compounds are consistent with previous reports [47,48,49], confirming the suitability of hydroethanolic solvents for extracting flavonoids and phenolic acids. Nonetheless, certain compounds tentatively identified in organic husks—such as 5-hydroxy-7,8-dimethoxyflavanone, methoxy-myricetin-3-*O*-hexoside, and a novel lignan (compound **25**)—have not been previously reported. In some cases, the identification between chromanes and carotenoids remains inconclusive (e.g., compounds **20** and **37**), requiring further structural elucidation to confirm their identity. The identification of extracted compounds is particularly relevant, as the high sensitivity and resolution of UPLC-ESI-QTOF-MS allow the detection of a wider range of molecules compared to conventional HPLC-DAD methods. In this study, in addition to phenolic compounds, additional constituents such as fatty acids (e.g., linoleic acid) and organic acids (e.g., uric acid) were tentatively identified. These compounds possess reducing properties that may interfere with spectrophotometric assays, potentially leading to an overestimation of TPC when using the Folin–Ciocalteu reagent [50]. It is important to note that all compound identifications were tentative, and no quantitative analysis was performed. Considering that walnut green husk is an agro-industrial by-product not originally intended for human consumption, further safety evaluations are essential. In particular, cytotoxicity assays are necessary to evaluate the potential health risks associated with these compounds, especially when the extracts are incorporated into food formulations.

### 3.3. Cytotoxic Effect of Extracts and Antioxidant Activity In Vivo

The cytotoxicity evaluation is a fundamental criterion for determining the safety of the obtained extract. Table 2 presents the results of the cell viability and cytotoxicity assays for extracts from both organic and traditional crops.

According to established toxicity classification criteria, extracts with a CC_50_ below 10 µg/mL are considered highly toxic, 11–30 µg/mL moderately toxic, 31–50 µg/mL slightly toxic, and non-toxic when CC_50_ exceeds 50 µg/mL [51]. None of the extracts analyzed in this study exhibited cytotoxicity (Table 2), and both were therefore classified as non-toxic, indicating their potential safety for use in food applications.

Additionally, the organic extract, characterized by higher TPC and antioxidant activity (Figure 1), was selected for further analysis. Cell viability assays performed on HepG2 (ATCC HB-8065, Atacama, Chile) and HUVECs (ATCC CRL-1730, Atacama, Chile) confirmed high viability at concentrations above 100 µg/mL, further supporting the safety profile of the extract for potential food applications. Although such analysis and results are required for all extracts obtained from agroindustrial by-products, residues, or waste, it is important to consider the potential presence of traces of these compounds that could cause adverse effects, including pesticide residues [15] or allergenic responses. While allergenicity was not specifically evaluated in this study, it is not expected, as nut-related allergic reactions are primarily associated with nut proteins [52], which were not detected in the extract (Table 1).

**Figure 3:**
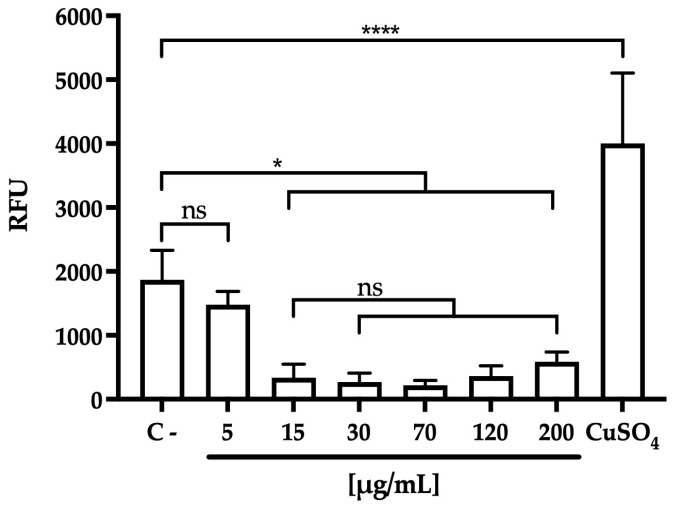
Reactive oxygen species (ROS) assay for organic extract at different concentrations in the Hep-G2 cell line over 4 h, expressed in relative fluorescence units (RFU). Untreated sample (C-), CuSO_4_ as positive control. Bar errors, indicate the standard deviation from the average of triplicates. ANOVA and Tukey post hoc tests indicate no significant differences (ns) or significant differences between samples at * *p* < 0.05, **** *p* < 0.0001.

To assess the in vitro antioxidant activity of polyphenols [9], intracellular ROS levels were measured in HepG2 cells treated with the organic extract (Figure 3). The results showed a significant reduction in ROS levels, confirming the strong antioxidant effect of the walnut green husk extract. No significant differences (*p* > 0.05) were observed across concentrations ranging from 15 to 200 µg/mL. These findings indicate that low-concentration extracts may require encapsulation to achieve an effective reduction in intracellular ROS. Future studies, including both in vitro and in vivo bioavailability methodology, must be conducted to fully evaluate the potential bioactivity of the encapsulated extract.

### 3.4. Effect of Encapsulant Concentration by Spray Drying

A polyphenol extract control without any wall material was spray-dried, resulting in no powder being obtained, which highlights the importance of using an encapsulation wall material for this technique.

A single-factor design was used to optimize chickpea protein concentration as an encapsulating agent in the spray-drying process. Based on preliminary findings, three concentrations (5%, 7.5%, and 10% *w*/*v*) were tested at a previously optimized inlet temperature of 136 °C. The resulting data are shown in Table 3.

As shown in Table 3, a chickpea protein concentration of 5% (*w*/*v*) was selected for encapsulating walnut green husk polyphenols, as it provided the highest TPC and AC values compared to 7.5% and 10% (*w*/*v*). No significant differences (*p* > 0.05) in encapsulation efficiency were observed across the tested concentrations. Although the 10% concentration produced a slightly lighter powder (L* = 88 ± 1), all samples were visually white. The slight but statistically significant decrease (*p* < 0.05) in TPC and AC values at the 10% concentration may be attributed to encapsulant overloading, matrix saturation, or dilution factor of antioxidant compounds, which could reduce observed activity. Considering the results, the 5% concentration was also preferred for economic and scalability reasons, as it minimizes protein use without compromising encapsulation performance.

### 3.5. Stability Analysis of Powder Additive at Different Relative Humidities

- *Total polyphenol content and antioxidant capacity*

Polyphenols are highly sensitive to environmental factors, including heat, humidity, light, and oxygen [9]. To evaluate their stability, the spray-dried powder, produced at 136 °C with 5% (*w*/*v*) chickpea protein, was stored under two relative humidity conditions (35% and 80% RH) for 21 days, with all samples kept in darkness to eliminate the effects of light.

**Figure 4:**
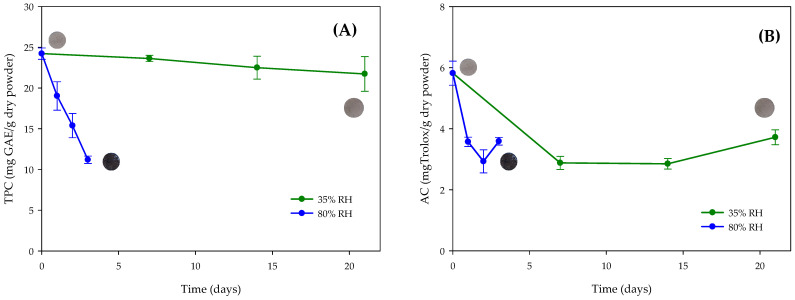
Polyphenol stability of spray-dried powder at 136 °C with 5% *w*/*v* encapsulating agent stored at different relative humidities (RH) for 21 days. (**A**) Total polyphenol content measured by Folin (TPC), (**B**) Antioxidant capacity (AC) measured by DPPH. Images inserted in (**A**,**B**) correspond to samples at the initial and final storage time to show visually change color. Bars indicate the standard deviation of triplicates. GAE = Gallic Acid Equivalent.

As shown in Figure 4A, the powder stored at 35% RH exhibited a gradual reduction in TPC values, with an approximately 10% loss over 21 days. In contrast, exposure to 80% RH resulted in a rapid decrease in TPC values from the first day, exceeding 50% by day 4, accompanied by unacceptable color changes that compromised its intended application, leading to discontinuation of measurements under this condition. Representative images of the powders at the beginning and end of storage are included in Figure 4. A similar trend was observed for antioxidant capacity (Figure 4B).

These findings are consistent with previous reports demonstrating the critical influence of humidity on polyphenol stability. Tovar [53] reported that antioxidant-rich powders, such as those derived from purple sweet potato (*Ipomoea batatas* L.), retain up to 85% of their antioxidant activity when stored under low-humidity conditions, whereas high humidity caused a loss of up to 60% within one week. In the present study, powders stored at low RH maintained 50% of their antioxidant activity after 21 days, while those at high RH lost 50% within just two days. Similar trends have been observed by Cáceres-Roa [54] for dry versus wet cocoa shell samples, and by Soto-Madrid [15] for walnut green husk extracts with chickpea protein additives when exposed to high humidity. López Fernández [55] also emphasized the critical role of relative humidity in the degradation of polyphenols and flavonoids in fruit and vegetable powders, ultimately reducing their antioxidant potential. Finally, these results confirm that moisture strongly compromises the stability of polyphenols from walnut green husk extracts.

- *Stability of color properties*

**Figure 5:**
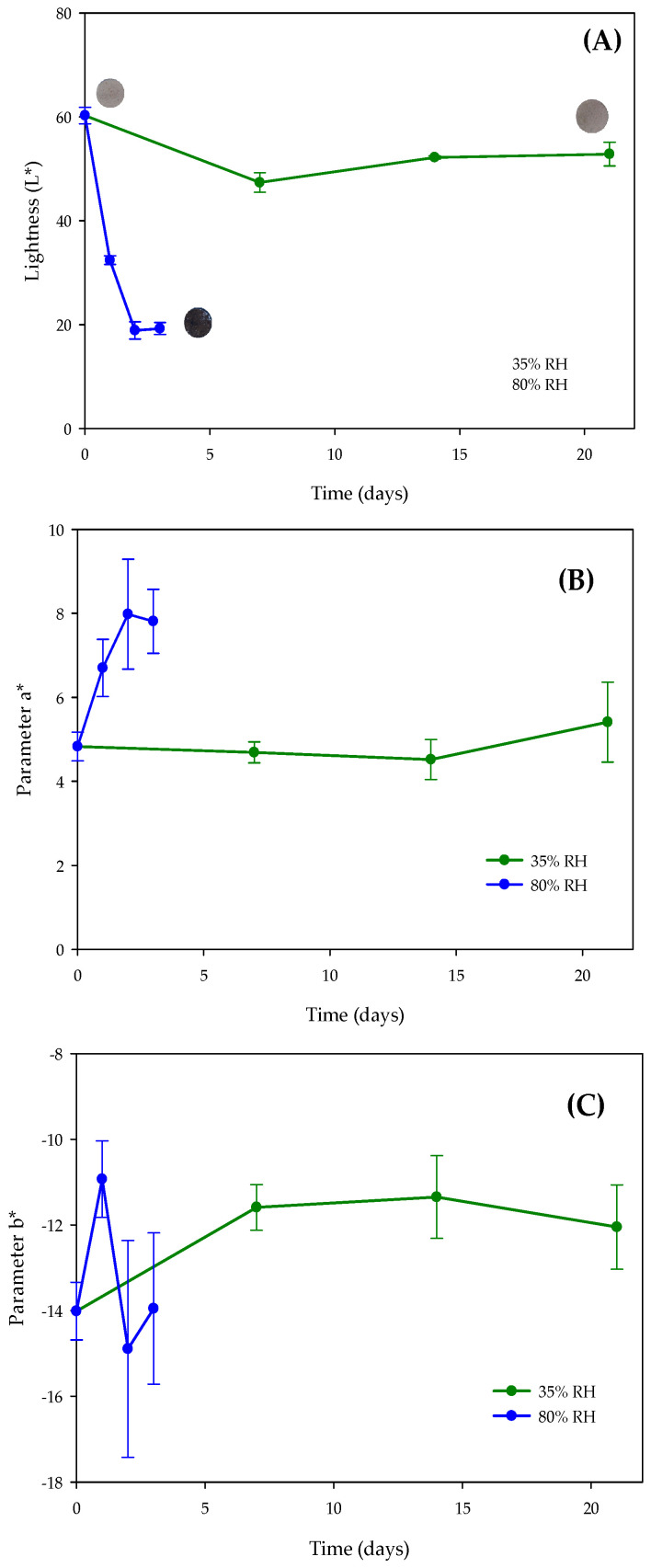
Stability of the color properties measured in the CIELAB space of the developed additive stored at different relative humidities (RH). (**A**) Lightness or L* parameter, (**B**) Parameter a* (red-green), (**C**) Parameter b* (blue-yellow). Images inserted in (**A**) correspond to samples at initial and final storage time to show visually change color.

Figure 5 illustrates the evolution of CIELAB color parameters (L*, a*, b*) during storage. Powders stored at low relative humidity (35% RH) exhibited minor decreases in all parameters over 21 days. In contrast, powders stored at high RH (80%) showed a rapid decrease in lightness (L*) and an increase in the a* value within the first two days, indicating a shift toward darker, brownish tones. This color change is attributed to polyphenol oxidation under humid conditions, which may negatively affect visual appeal and consumer acceptance. These observations align with previous studies by Soto-Madrid [15] and Tormos [56] (2022), which also reported reduced color stability and lightness in freeze-dried powders exposed to high humidity.

The browning observed at 80% RH is likely due to the oxidative reaction of polyphenol. A strong negative correlation was found between lightness (L*) and antioxidant capacity under these conditions (Pearson coefficient = 0.957), suggesting that polyphenol oxidation contributes to both color darkening and functional loss [57]. Based on these results, the optimal storage condition for maintaining powder stability is at low relative humidity (35%) and room temperature (25 °C), where color retention is positively correlated with antioxidant capacity.

### 3.6. Morphology Characterization of the Powder Additive

**Figure 6:**
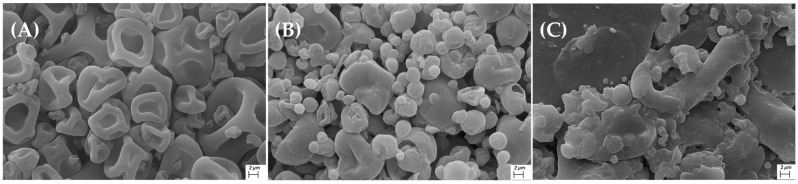
SEM of the powder additive developed from walnut green husk and chickpea protein. (**A**) Spray-dried chickpea protein control; (**B**) spray-dried additive at zero time; and (**C**) spray-dried additive stored at 35% RH after 21 days. Mag. 10.00 K X.

Scanning electron microscopy (SEM) was employed to evaluate the morphology of the natural powder additive. The micrograph of the CPP control (Figure 6A) displays empty spherical structures of varying sizes with wrinkled surfaces, characteristic of protein spray drying [58]. In contrast, Figure 6B reveals more defined, smooth spherical structures of diverse sizes, indicating successful encapsulation of phenolic compounds by the protein. Additionally, empty and larger structures similar to those in the CPP control were observed, indicating that a 5% *w*/*v* CPP concentration is sufficient when the protein is dried alone.

Figure 6C presents the sample stored under controlled relative humidity (35%) for 21 days. To facilitate analysis, the sample was compressed into tablets with a diameter of 0.5 cm, which accounts for their flattened appearance. Despite this processing, the spherical structures encapsulating phenolic compounds from the walnut green husk remained clearly identifiable, in agreement with previously reported antioxidant activity. Notably, this activity remained stable for 14 days, indicating that the extract retained partial stability within the protein matrix during storage.

## 4. Conclusions

A polyphenolic extract was successfully obtained from walnut green husk using ultrasound-assisted extraction, demonstrating non-toxicity and showing no dependence on pesticide residues or cultivation methods (organic or traditional). Extracts from organic samples exhibited a higher total polyphenol content (TPC) and antioxidant activity, with 37 compounds identified compared to 22 in the traditional samples. These findings demonstrated that walnut green husk represents a promising source for the development of natural antioxidant additives. Future research should incorporate complementary analytical methods to confirm tentative compound identifications and validate quantitative assessments. The isolation of individual compounds through chromatographic techniques, followed by Nuclear Magnetic Resonance (NMR) spectroscopy, is highly recommended for definitive structural elucidation. In addition, high-sensitivity and high-throughput quantification will require Liquid Chromatography-Tandem Mass Spectrometry (LC-MS/MS) and/or High-Performance Liquid Chromatography with Diode Array Detection (HPLC-DAD), using calibration curves of analytical standards.

A sustainable and vegan antioxidant additive was developed by encapsulating walnut green husk extract within chickpea protein matrices. The optimal formulation, obtained with 5% (*w*/*v*) chickpea protein spray-dried at 136 °C, exhibited the highest polyphenol content and antioxidant capacity. Storage under low relative humidity (≤35%) is recommended to preserve the antioxidant stability at room temperature. Furthermore, a clear relationship was observed between powder lightness and activity, indicating that darker powders had a lower antioxidant capacity.

These findings highlight the potential of plant-based protein polymers to stabilize polyphenol-rich extracts derived from agro-industrial by-products. The proposed system represents a promising alternative to synthetic and animal-derived antioxidants in the food industry, combining sustainability with technological feasibility. Cytotoxicity tests confirmed the safety of the formulation, while stability studies provided evidence of its practical viability. Future studies could be structured to clarify bioavailability within in vivo experiments. Several factors influence a substance’s bioavailability, including the method of delivery, interactions with other substances, absorption, liver processing, and elimination.

The results demonstrate the strong sensitivity of polyphenols and antioxidant capacity to moisture, given its critical role in the shelf life of the powdered product. Stability testing under varying humidity conditions proved highly relevant for practical storage and applications, and the influence of temperature must also be considered to ensure long-term integrity under both storage and food processing conditions

Further research recommendations should evaluate the functional properties and storage stability of this vegan antioxidant additive when applied in real food systems, including comprehensive shelf-life studies, sensory evaluation, and consumer acceptance trials. Application in dairy alternative products represents one example of its potential use; however, claims validation across diverse food matrices is required to confirm its technological performance. Such investigations are essential for advancing the development of innovative, sustainable ingredients tailored to modern food systems.

## Figures and Tables

**Table 1:** Proximal analysis of the husk and aqueous extract of walnut green husk obtained from organic and traditional crops.

Analysis	Organic Sample		Traditional Sample ^2^	
	Green Husk	Aqueous Extract	Green Husk	Aqueous Extract
	(g/100 g Dry Base)	(g/100 g Wet Base)	(g/100 g Dry Base)	(g/100 g Wet Base)
Moisture	8.30 ± 0.01	94.40 ± 0.01	7.50 ± 0.01	93.90 ± 0.01
Proteins (%N × 5.3 ^1^)	4.0 ± 0.2	* ND	7.0 ± 0.2	* ND
Lipids	1.9 ± 0.1	** ND	1.9 ± 0.1	** ND
Ash	16.70 ± 0.05	2.00 ± 0.04	12.6 ± 0.05	1.00 ± 0.06
Crude Fiber	19 ± 1	*** ND	20 ± 1	*** ND
Nitrogen-free extract (N.N.E.)	51 ± 1	3.6 ± 0.08	51 ± 1	5.00 ± 0.08

ND: Not detected with a limit of detection of * ≤ 0.39 g/100 g; ** ≤ 0.52 g/100 g; *** ≤ 0.59 g/100 g. ^1^ Conversion factor for nuts [36]. ^2^ Data obtained from Soto-Madrid [15].

**Table 2:** Cytotoxicity assay results for traditional and organic samples. CC_50_ values represent the concentrations that inhibit 50% of cell proliferation. The same letter (^a^) indicates non-significant differences (*p* > 0.05) between samples.

Crop	CC50 [μg/mL]	Cytotoxicity
Organic	96 ± 2 ^a^	Non-toxic
Traditional	90 ± 9 ^a^	Non-toxic

**Table 3:** Results of response variables of factorial design of the additive dried at 136 °C at 3 different protein concentrations.

Response Variable	5% *w*/*v*	7.5% *w*/*v*	10% *w*/*v*
TPC (mg GAE/g dry sample)	26.9 ^a^ ± 1.8	23.9 ^b^ ± 0.5	25.0 ^b^ ± 0.5
AC (mg Trolox/g of dry sample)	10.4 ^a^ ± 0.5	8.0 ^b^ ± 0.5	7.1 ^c^ ± 0.4
Moisture (% dry basis)	5.5 ^a^ ± 0.4	5.3 ^a^ ± 0.4	5.2 ^a^ ± 0.4
a_w_	0.21 ^a^ ± 0.01	0.22 ^a^ ± 0.02	0.28 ^b^ ± 0.01
Encapsulation efficiency (%)	52 ^a^ ± 5	50 ^a^ ± 2	52 ^a^ ± 1
Yield (%)	52	54	66
Color Parameter L*	80 ^c^ ± 3	86 ^b^ ± 2	88 ^a^ ± 1
Color Parameter a*	3 ^b^ ± 1	3 ^a^ ± 1	4 ^a^ ± 1
Color Parameter b*	−15 ^c^ ± 2	−20 ^b^ ± 1	−21 ^a^ ± 1

TPC: Total polyphenol content; AC: Antioxidant capacity measured by DPPH. GAE: Gallic Acid Equivalent. Values are expressed as mean ± standard deviation (*n* = 3). Different letters (a,b,c) indicate significant differences between columns (*p* < 0.05).

## Data Availability

The raw data supporting the conclusions of this article will be made available by the authors on request.

## Supplementary Materials

The following supporting information can be downloaded at: https://www.mdpi.com/article/10.3390/polym17172371/s1, Figure S1. Location Map. Table S1: Caption.

## Abbreviations

The following abbreviations are used in this manuscript:

AC	Antioxidant capacity
AMSL	Above mean sea level
ANOVA	Analysis of variance
CC50	Concentrations that inhibit 50% of cell proliferation
CIEDE2000	Color-difference formula
CPP	Chickpea protein
DCFH-DA	2′,7′-dichlorofluorescein diacetate assay
DPPH	2,2-diphenyl-1-picrylhydrazyl
GAE	Gallic acid equivalent
HPLC-DAD	High-Performance Liquid Chromatography with Diode Array Detection
ND	Not detected
RFU	Relative fluorescence units
RH	Relative humidity
ROS	Intracellular reactive oxygen species
SEM	Scanning Electron Microscopy
TPC	Total polyphenol content
TSS	Total soluble solids
UPLC-QTOF-ESI-MS	Ultra-performance liquid chromatography quadrupole time-of-flight electrospray ionization mass spectrometry
