# Supplementary material for: Upcycling Walnut Green Husk: Polyphenol-Rich Extracts from Traditional vs. Organic Crops for Spray-Dried Vegan Additive Development
Source: Polymers (Basel). 2025 Aug 31;17(17):2371. doi: 10.3390/polym17172371 (PMC12431422; doi:10.3390/polym17172371)
Supplement: Supplementary file 1 [file polymers-17-02371-s001.zip › Table S1.pdf]

N°	Retention time (min)	Formula	Measured mass *m/z*	Error (ppm)	Tentative compound identification	Classification	References
1	6.90	$C_7H_6O_5$	169.0140	-1.35	Gallic acid	Phenolic acids	Soto Madrid, 2024; Sheng et al., 2021; Kithi et al., 2023
2	8.27	$C_{17}H_{16}O_5$	321.0730	-4.67	5-Hydroxy-7,8-dimethoxyflavanone	Flavonoids	not reported
3	8.41	$C_9H_8O_4$	179.0347	-1.63	Caffeic acid	Cinnamic acids and derivatives	Soto Madrid, 2024; Sheng et al., 2021; Kithi et al., 2023
4	8.50	$C_7H_6O_4$	153.0186	-4.81	Protocatechuic acid	Phenolic acids	Soto Madrid, 2024; Sheng et al., 2021; Kithi et al., 2023; Wu et al, 2021
5	8.70	$C_9H_6O_3$	161.0210	-2.83	7-hydroxy-coumarin	Coumarins	Sheng et al., 2021
6	8.74	$C_{30}H_{26}O_{12}$	577.1357	0.98	Procyanidin B2	Flavonoids	Wu et al., 2021
7	8.78	$C_{15}H_{18}O_8$	325.0943	4.52	1-O-[(2*E*)-3-(2-hydroxyphenyl)-1-oxo-2-propen-1-yl]-beta-D-glucopyranose	Cinnamic acids and derivatives	not reported
8	8.98	$C_8H_8O_5$	183.0294	-2.53	Dihydroxymandelic acid	Cinnamic acids and derivatives	Sheng et al., 2023
9	9.51	$C_{15}H_{14}O_6$	289.0720	0.95	Catechin	Flavonoids	Soto Madrid, 2024; Sheng et al., 2021; Kithi et al., 2023
	9.51	$C_{15}H_{14}O_6$	289.0720	0.95	(-)-Epicatechin	Flavonoids	Kithi et al., 2023; Wu et al., 2021
	9.51	$C_{15}H_{14}O_6$	289.0720	0.95	(+)-Catechin	Flavonoids	Kithi et al., 2023; Wu et al., 2021; Jahanban-Esfahlan et al., 2023
10	9.53	$C_9H_8O_3$	163.0396	-3.10	*p*-Coumaric acid	Cinnamic acids and derivatives	Amic et al., 2021; Sheng et al., 2023; Kithi et al., 2023
11	9.85	$C_{10}H_{12}O_4$	195.0655	-3.83	Benzoic acid	Phenolic acids	Jahanban-Esfahlan et al., 2023
12	9.90	$C_{10}H_{10}O_4$	193.0504	-1.03	*trans*-Ferulic acid	Phenolic acids	Sheng et al., 2023; Jahanban-Esfahlan et al., 2023
13	10.01	$C_{15}H_{18}O_7$	309.0980	0.14	1-*O*-*trans*-cinnamoyl-beta-D-glucopyranose	Cinnamic acids and derivatives	not reported
14	10.48	$C_{16}H_{20}O_{10}$	393.0817	3.70	3-(benzoyloxy)-2-hydroxypropyl beta-D-glucopyranosiduronic acid	Coumarins	not reported

15	10.71	$C_{16}H_{18}O_8$	337.0930	0.40	3-*p*-Coumaroylquinic acid	Phenolic acids	Vieira et al., 2020; Amic et al., 2021
16	10.80	$C_7H_6O_4$	153.0189	-2.83	2,5-Dihydroxybenzoic acid	Phenolic acids	Kithi et al., 2023
17	11.02	$C_9H_8O_2$	147.0447	-3.04	Cinnamic acid	Cinnamic acids and derivatives	Wu et al., 2021
18	11.17	$C_{21}H_{20}O_{13}$	479.0829	-0.48	Myricetin-*O*-hexoside	Flavonoids	Sheng et al., 2021
19	11.24	$C_{16}H_{18}O_9$	375.0685	-3.42	Chlorogenic acid	Cinnamic acids and derivatives	Jahanban-Esfahlan et al., 2023
20	11.44	$C_{21}H_{24}O_{11}$	451.1252	1.46	3*R*,.3'*R*,4*R*,6'*S*,7*R*)-5-Hydroxy-6',7-dimethyl-6,8-dioxo-3',4,4',5',6,6',7,8-octahydrospiro[isochromene-3,2'-pyran]-3',4,7-triyl triacetate	Chromanes	not reported
	11.44	$C_{21}H_{24}O_{11}$	451.1252	1.46	Roccellin	Chromanes	not reported
21	11.83	$C_{11}H_{12}O_5$	229.0499	7.82	Sinapic acid	Coumarins	Sheng et al., 2023
22	11.99	$C_{21}H_{20}O_{12}$	463.0876	-1.30	Quercetin-3-*O*-hexoside	Flavonoids	Vieira et al., 2020
	11.99	$C_{21}H_{20}O_{12}$	463.0876	-1.30	Myricitrin	Flavonoids	Sheng et al., 2021
23	12.05	$C_{14}H_6O_8$	300.9985	-1.63	Ellagic acid	Lignans	Wu et al., 2021
24	12.17	$C_{22}H_{18}O_{10}$	441.0835	1.81	(-)-Epicatechin gallate	Flavonoids	Wu et al., 2021
25	12.34	$C_{23}H_{30}O_{11}$	503.1558	4.92	3-hydroxy-1.2-bis(4-hydroxy-3-methoxyphenyl)propylHexopyranoside.	Lignans	not reported
26	12.480	$C_{15}H_{10}O_6$	285.0404	-0,20	Kaempferol	Flavonoids	Sheng et al., 2021
	12.480	$C_{15}H_{10}O_6$	285.0404	-0,20	Luteolin	Flavonoids	Wu et al., 2021
27	12.480	$C_{15}H_{12}O_7$	303.051	-0,20	Dihydroquercetin	Flavonoids	Wu et al., 2021
	12.480	$C_{15}H_{12}O_7$	303.051	0,20	Taxifolin	Flavonoids	Sheng et al., 2023
28	12.56	$C_{20}H_{18}O_{11}$	433.0772	0,28	Quercetin-3-*O*-pentoside	Flavonoids	Sheng et al., 2021; Vieira et al., 2020
29	12.73	$C_{20}H_{18}O_{12}$	449.0767	-1.00	Myricetin-3-*O*-pentoside	Flavonoids	Medic et al., 2021
30	12.82	$C_{23}H_{22}O_{13}$	527.0769	-7.46	Quercetin 3-*O*-(6-*O*-acetyl-beta-D-glucopyranoside)	Flavonoids	Wu et al., 2021

31	12.85	$C_{21}H_{20}O_{11}$	447.0923	-1.79	Quercetin 3-*O*-rhamnoside	Flavonoids	Medic et al., 2021
32	12.85	$C_{22}H_{22}O_{13}$	515.0827	-0.38	Methoxy-myricetin-3-*O*-hexoside	Flavonoids	not reported
33	13.22	$C_9H_8O_2$	147.0451	4.50	Cinnamic acid	Cinnamic acids and derivatives	Wu et al., 2021
34	13.50	$C_{15}H_{18}O_8$	325.0944	-7.46	*p*-Coumaric acid glycoside	Cinnamic acids and derivatives	Jahanban-Esfahlan et al., 2019
35	13.60	$C_{25}H_{28}O_{12}$	519.1506	-0.41	(2*E*)-3-(2-{[6-*O*-(3.4.5-Trimethoxybenzoyl)-beta-D-glucopyranosyl]oxy}phenyl)acrylic acid	Cinnamic acids and derivatives	not reported
36	16.75	$C_{15}H_{12}O_5$	271.0616	1.33	Naringenin	Flavonoids	Wu et al., 2021; Medic et al., 2021; Sheng et al., 2023
37	17.99	$C_{13}H_{22}O_4$	241.1449	1.39	(3*S*,5*R*,6*R*,7*E*)-3,5,6-Trihydroxy-7-megastigmen-9-one	Carotenoids	not reported
	17.99	$C_{13}H_{22}O_4$	241.1449	1.39	8-[(*E*)-3-hydroxybut-1-enyl]-1,5-dimethyl-6-oxabicyclo[3.2.1]octane-3,8-diol	Carotenoids	not reported
