# Supplementary figures and images for: Upcycling Walnut Green Husk: Polyphenol-Rich Extracts from Traditional vs. Organic Crops for Spray-Dried Vegan Additive Development
Source: Polymers (Basel). 2025 Aug 31;17(17):2371. doi: 10.3390/polym17172371 (PMC12431422; doi:10.3390/polym17172371)

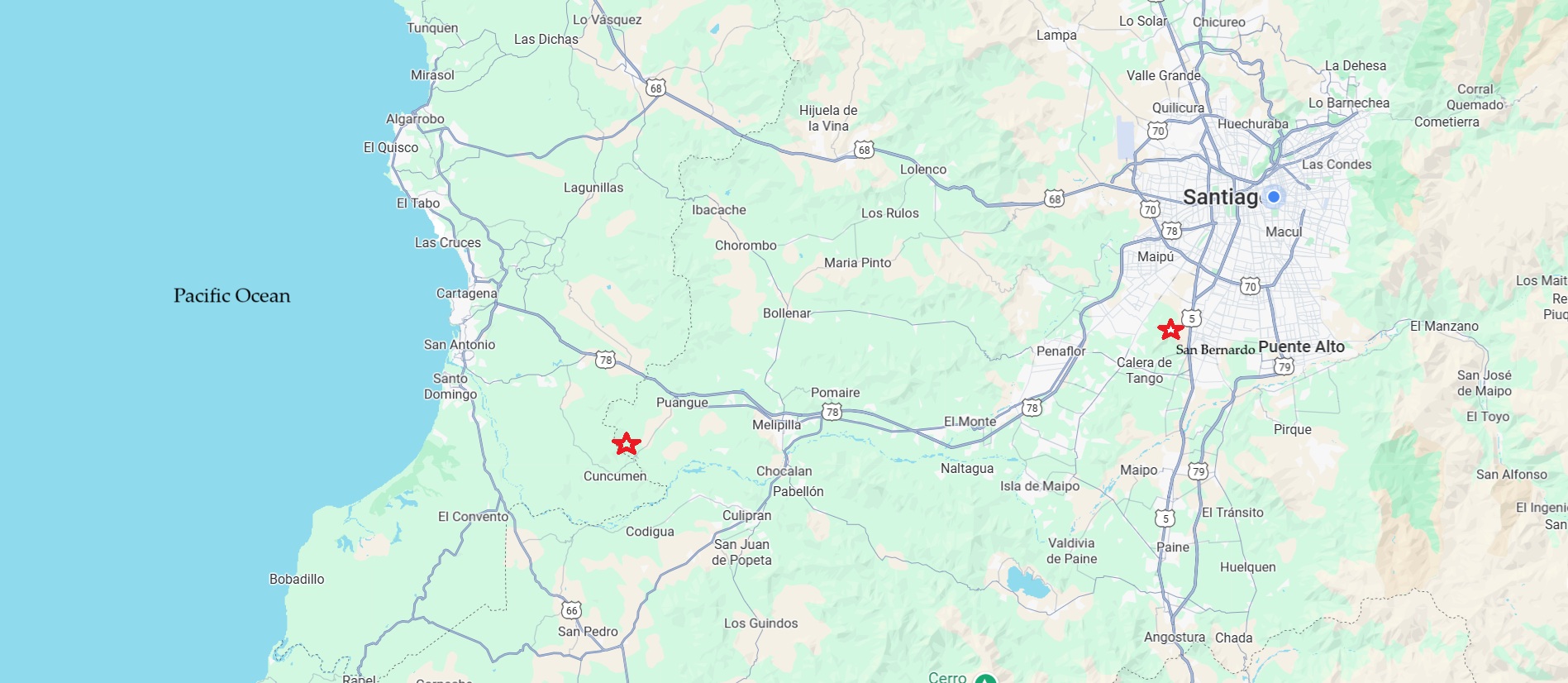

Supplement: Supplementary file 1 [file polymers-17-02371-s001.zip › Figure S1. Location Map.jpg]
